# Lymphatic–venous anastomosis: Cracking the code of Alzheimer’s disease treatment?

**DOI:** 10.4103/NRR.NRR-D-25-00540

**Published:** 2025-09-03

**Authors:** Fan Fan, Nannan Zhao, Mian Guo

**Affiliations:** Department of Physiology, Medical College of Georgia, Augusta University, Augusta, GA, USA; Department of Neurosurgery, the Second Affiliated Hospital of Harbin Medical University, Harbin, Heilongjiang Province, China

**Figure 1:**
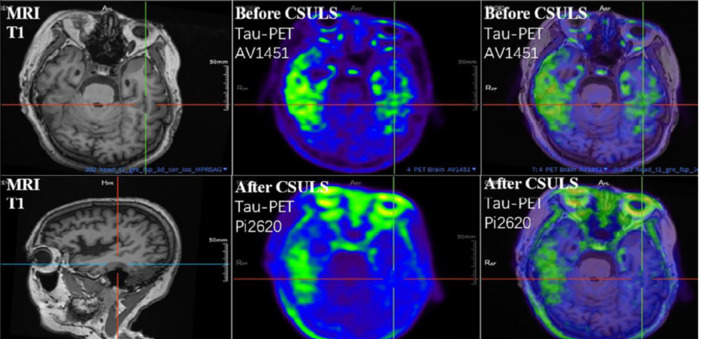
Lymphatic-venous anastomosis reduced tau accumulation in Alzheimer’s disease brain. Cervical Shunting to Unclog Cerebral Lymphatic Systems (CSULS) reduced overall brain tau accumulation in an Alzheimer’s disease patient, especially in the left temporal lobe, as detected by a tau-positron emission tomography (PET) scan. Reprinted with permission from Li et al. (2024).

Deep cervical lymph–venous anastomosis (LVA) is a surgical procedure initially developed to treat cervical lymphatic obstruction, such as lymphedema, a condition caused by the accumulation of lymphatic fluid due to blocked or damaged lymphatic vessels. In early 2024, Dr. Qingping Xie from Hangzhou Qiushi Hospital, China, and Dr. Wei F. Chen from the Cleveland Clinic, USA, adapted LVA for the treatment of patients with Alzheimer’s disease (AD). As a VIEWPOINT, they presented a video showcasing the post-surgery cognitive recovery of an 84-year-old AD patient (Xie et al., 2024). Since then, many Chinese hospitals have initiated clinical trials for the treatment of AD (e.g., ChiCTR2400084617, NCT06530732, and NCT06448442) and type 2 diabetes combined with AD (ChiCTR2400093030) using LVA. In June 2024, a letter by Li et al. (2024) demonstrated similar beneficial cognitive effects using a slightly different Cervical Shunting to Unclog Cerebral Lymphatic Systems (CSULS) surgical procedure in AD patients. In a 5-week post-surgery follow-up study of an AD patient who met the diagnostic criteria set by the National Institute on Aging-Alzheimer’s Association, improvements in cognitive function were confirmed. Furthermore, overall brain tau accumulation was reduced (**Figure 1**), and a significant enhancement in brain glucose metabolism was observed in this patient (Li et al., 2024). More recently, Dr. Mian Guo and team initiated a multicenter, prospective clinical study (ChiCTR2500095309) on LVA for the treatment of moderate to severe AD. To date, the LVA surgical approach has been adopted by over 100 hospitals in China, with thousands of AD patients undergoing the treatment. Many of these patients have reported improvements in AD symptoms on social media. Although anecdotal reports of symptom improvement are widespread on social media, robust analytical data remain limited. As an exploratory approach, LVA has demonstrated explosive growth and continues to attract considerable attention. However, published studies to date have primarily focused on Aβ clearance. This perspective is distinct in that it emphasizes the potential vascular changes that may underlie or modulate the therapeutic effects of LVA.

Is LVA truly cracking the code for AD treatment, or is it just a fantastic fairy tale?

AD is a neurodegenerative disorder characterized by progressive cognitive decline. Although the precise mechanisms of AD are not fully understood, especially given the current lack of effective treatments, the amyloid-beta (Aβ) cascade hypothesis remains a leading theory. This hypothesis suggests that excessive Aβ deposition triggers a cascade of pathophysiological events, including tau hyperphosphorylation, neurofibrillary tangle formation that impairs microtubule function in neurons, and subsequent neurodegeneration, leading to cognitive decline. Aβ accumulation in the brain occurs when there is an imbalance between the production and clearance of Aβ. Genetic factors significantly contribute to this imbalance. In early-onset familial AD, mutations in genes such as presenilin 1, a crucial component of γ-secretase, disrupt the processing of amyloid precursor protein. This alteration leads to an increased production of the more aggregation-prone Aβ_42_ form, which is a key driver of amyloid plaque formation. In late-onset AD, individuals carrying the APOEε4 allele are at a higher risk of developing the disease. This increased risk is believed to be linked to the impaired clearance of Aβ from the extracellular space, contributing to the accumulation of amyloid plaques in the brain. Other factors, such as oxidative stress, inflammation, aging, small vessel diseases, metabolic syndromes, Down syndrome, and sleep deprivation, all contribute to the imbalance of Aβ production and clearance. In 2021, the US Food and Drug Administration approved aducanumab, the first Aβ monoclonal antibody, for the treatment of AD. This approval represented a major shift in AD therapy, moving from symptomatic management with acetylcholinesterase inhibitors and N-methyl-D-aspartic acid receptor antagonists to targeting Aβ clearance. To date, the US Food and Drug Administration has approved three Aβ antibodies for AD: aducanumab, lecanemab, and donanemab (Mullard, 2024). However, aducanumab was discontinued in January 2024 by Biogen due to business considerations and a decision to reprioritize resources in AD research, focusing more on lecanemab, although other concerns also contributed to this decision. Despite anti-Aβ therapies showing some effectiveness in clearing plaques and delaying cognitive decline in clinical studies, about 40% of treated patients have developed amyloid-related imaging abnormalities. Additionally, individuals carrying the APOEε4 variant face a significantly higher risk of cerebral edema and hemorrhage, the two major symptoms of amyloid-related imaging abnormalities. This is believed to result from the mobilization of amyloid plaques, along with inflammation and vascular damage (Cogswell et al., 2022). These findings suggest that an exclusive focus on the Aβ cascade hypothesis in AD treatment may overlook other critical multifactorial contributors. This implies the importance of considering additional AD causal factors, such as vascular dysfunction.

Brain hypoperfusion is an early symptom of AD. The Fan team conducted a longitudinal characterization of cerebral hemodynamics in the TgF344-AD rat model of AD (Fang et al., 2023). They found that brain hypoperfusion, associated with impaired cerebral blood flow autoregulation, disrupted myogenic response, increased blood-brain barrier permeability, and neurovascular uncoupling, emerged at 4 months of age. This occurred two months prior to the appearance of Aβ plaques and the onset of cognitive deficits in this well-established AD model. Moreover, they found that the contractility of cerebral vascular smooth muscle cells (VSMC) isolated from this AD rat was reduced, and Aβ-treated cerebral VSMCs isolated from wild-type rats also displayed reduced contractility (Fang et al., 2023). Transcriptomic profiling analysis revealed that pathways essential for regulating cellular contraction, oxidative stress, and inflammation are significantly altered in primary cerebral VSMCs from AD rats compared to F344 control rats (Tang et al., 2025). Furthermore, the Attwell team reported that Aβ constricts pericytes in human AD cerebral capillaries, which contributes to brain hypoperfusion (Nortley et al., 2019). This evidence suggests a vicious cycle between Aβ abnormalities and reduced cerebral perfusion, indicating that targeting not only Aβ but also the combined effects of Aβ and vascular contributions to AD pathogenesis may offer a more comprehensive approach to AD treatment.

In the brain, mechanisms involved in Aβ clearance include both enzymatic and non-enzymatic pathways (Tarasoff-Conway et al., 2015). Neprilysin and insulin-degrading enzyme are important enzymes involved in the degradation of Aβ peptides, thereby reducing Aβ accumulation in the brain. Aβ uptake through phagocytosis by glial and VSMCs also plays an essential role in Aβ clearance. Additionally, facilitated by low-density lipoprotein receptor-related protein 1, brain Aβ can cross the blood-brain barrier into the bloodstream. Pericytes not only regulate cerebral blood flow and support blood–brain barrier integrity but also facilitate Aβ clearance (Tarasoff-Conway et al., 2015). Conversely, infiltrated peripheral immune cells, including myeloid cells, contribute to Aβ clearance in the brain cortex and on the cerebral vascular wall. Moreover, the glymphatic system facilitates the removal of Aβ from the brain via the interstitial fluid into the cerebrospinal fluid. Aβ is then transported via the meningeal lymphatic vessels to the deep cervical lymph nodes and eventually drains into the bloodstream via the lymphatic system, where Aβ is eliminated by the liver and kidneys (Iliff et al., 2012; Louveau et al., 2015). The glymphatic system, therefore, links Aβ clearance to the bloodstream, potentially impacting cerebral vascular function in AD.

**Figure 2:**
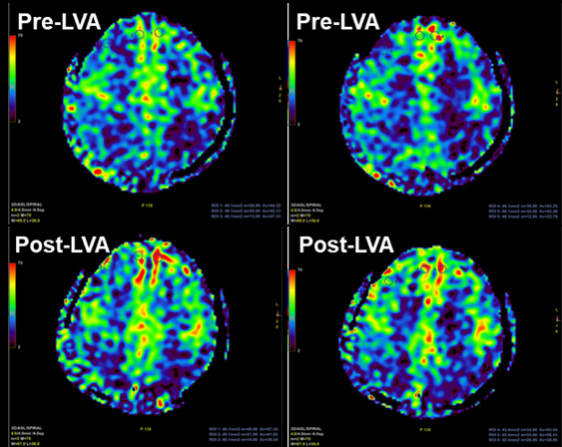
Lymphatic-venous anastomosis improved brain perfusion in Alzheimer’s disease. Cerebral perfusion in an Alzheimer’s disease patient was enhanced 7 days after undergoing lymph–venous anastomosis, as detected and quantified using arterial spin labeling magnetic resonance imaging with spiral acquisition on a three-dimensional scanner. Sourced from Dr. Mian Guo’s research team.

The glymphatic system, discovered by the Nedergaard team (Iliff et al., 2012), is a brain-wide waste clearance pathway that operates through a specialized network of perivascular spaces. Functionally analogous to the peripheral lymphatic system, it is influenced by factors such as vascular pulsation, breathing, sleep cycles, and the water channel protein Aquaporin 4 (Iliff et al., 2012; Louveau et al., 2015). Since vascular pulsation is essential in driving the movement of cerebrospinal fluid and interstitial fluid within the brain, the impaired myogenic response and cerebral blood flow autoregulation observed in AD (Fang et al., 2023; Tang et al., 2025) are likely linked to reduced Aβ clearance via the glymphatic system. Additionally, inflammatory pathways activated in AD, as identified through VASM transcriptomic profiling (Tang et al., 2025), are likely contributing to impaired glymphatic drainage, which is further exacerbated by the enlarged perivascular spaces commonly observed in AD (Tarasoff-Conway et al., 2015). Therefore, LVA that enhances glymphatic system drainage may not only improve Aβ clearance via the glymphatic system but also restore vascular function and enhance brain perfusion by mitigating Aβ-induced VSMC contractility. Following this line, the Guo team discovered that brain perfusion, measured by arterial spin labeling magnetic resonance imaging using spiral acquisition on a 3D scanner and quantified through signal intensities, was significantly increased in an AD patient 7 days after LVA (**Figure 2**). This evidence provides preliminary support that LVA may offer a promising therapeutic avenue by simultaneously targeting impaired glymphatic clearance and cerebrovascular dysfunction in AD.

Although LVA has brought hope with improvements in cognition, brain perfusion, and tau burden to AD patients, numerous critical questions remain regarding its underlying mechanisms, long-term efficacy, and optimal clinical application. (1) To date, only limited robust analytical data have been published, mainly because this surgical approach is relatively new in the context of AD. (2) Patient enrollment criteria lack standardization. Factors such as age, sex, medical history, and genetic background have yet to be systematically categorized or analyzed through subgrouping. Most of the patients undergoing surgery are in the middle to late stages and have severe cognitive impairment; some even have impaired mobility. Although it is difficult for some of these patients to undergo regular imaging examinations in hospitals, alternative methods of monitoring and assessment could be explored to provide more consistent and frequent evaluations. (3) AD is a progressive neurodegenerative condition, and promoting neuroregeneration remains one of the most challenging goals in the field. This raises a critical question: How can LVA lead to measurable improvements in cognition and brain perfusion as early as one-week post-surgery? Given the complexity of neuronal repair, one would typically expect a much longer timeframe for cognitive recovery following enhanced Aβ clearance. Moreover, neuronal loss presents a further challenge, as current interventions cannot revive dead neurons, only potentially support the function of surviving ones or prevent further degeneration. Considering that delirium is a common postoperative side effect following LVA, is it possible that the acute improvement in cognition, particularly in attention and reaction speed, is merely a “side effect” or a “brain reset” induced by anesthesia rather than a direct result of the surgery itself? (4) Conversely, if the glymphatic system has been obstructed in AD patients prior to LVA, elevated pressure and volume in the perivascular spaces, choroid plexus, and cerebral ventricles may occur. These pressure elevations could compress blood vessels and brain parenchyma, impairing cerebral blood flow autoregulation and leading to hypoperfusion and neuronal damage. Upon sudden release of this pressure, cerebral hypoperfusion may improve almost immediately, which could explain the acute enhancement in cognition and brain perfusion observed shortly after surgery. (5) From LVA performed by the Xie team (Xie et al., 2024) to CSULS introduced by Li et al. (2024) to the deep cervical lymph nodes-internal jugular vein anastomosis developed by the Guo team, although all follow the same overarching concept of enhancing glymphatic drainage, the surgical procedures themselves are somewhat different. Whether these variations in surgical technique impact the efficacy and long-term outcomes of glymphatic drainage remains unclear and warrants further investigation. (6) As with any surgical procedure, LVA carries potential risks such as infection, lymphorrhea, thrombosis, anastomotic failure, and venous hypertension. Furthermore, due to the anatomical proximity of the surgical site to autonomic nervous structures, it remains unclear whether neck surgery influences autonomic nervous system function or contributes to cognitive improvement in patients with AD. (7) The improved Aβ clearance through LVA ultimately enters the bloodstream and is removed by the liver and kidneys (Iliff et al., 2012; Louveau et al., 2015). Therefore, whether the efficacy of this surgical intervention might be altered in patients with hepatic or renal dysfunction should also be considered.

In summary, LVA offers a promising new avenue for the treatment of AD. However, its efficacy and underlying mechanisms remain to be fully elucidated. Moreover, to date, all LVA-related studies have focused exclusively on Aβ clearance, with no investigation into the vascular changes that may underlie or influence its effects. To clarify these issues, well-designed longitudinal clinical studies with standardized enrollment criteria and mechanistic research using animal models are necessary. Until such evidence is available, cautious interpretation of preliminary findings is warranted, and regulatory oversight of related clinical trials remains essential. We hope that LVA is more than just an intriguing story and becomes a transformative breakthrough in AD therapy. If proven true, it could mark a paradigm shift, moving AD treatment beyond Aβ clearance alone into a new era that equally targets vascular health and glymphatic restoration.

*This work was supported by AG057842 from the National Institutes of Health, TRIBA/Physiology Faculty Startup Fund from Augusta University (to FF), and the National Natural Science Foundation of China (82173384) (to MG).*
